# Rapid detection of *mexX* in *Pseudomonas aeruginosa* based on CRISPR-Cas13a coupled with recombinase polymerase amplification

**DOI:** 10.3389/fmicb.2024.1341179

**Published:** 2024-01-31

**Authors:** Xiao-Xuan Zhu, Ying-Si Wang, Su-Juan Li, Ru-Qun Peng, Xia Wen, Hong Peng, Qing-Shan Shi, Gang Zhou, Xiao-Bao Xie, Jie Wang

**Affiliations:** ^1^Guangdong Provincial Key Laboratory of Nutraceuticals and Functional Foods, College of Food Science, South China Agricultural University, Guangzhou, Guangdong, China; ^2^Guangdong Provincial Key Laboratory of Microbial Culture Collection and Application, State Key Laboratory of Applied Microbiology Southern China, Institute of Microbiology, Guangdong Academy of Sciences, Guangzhou, Guangdong, China

**Keywords:** *Pseudomonas aeruginosa*, MexX, Crispr-cas13a, recombinase polymerase amplification, detection

## Abstract

The principal pathogen responsible for chronic urinary tract infections, immunocompromised hosts, and cystic fibrosis patients is *Pseudomonas aeruginosa*, which is difficult to eradicate. Due to the extensive use of antibiotics, multidrug-resistant *P. aeruginosa* has evolved, complicating clinical therapy. Therefore, a rapid and efficient approach for detecting *P. aeruginosa* strains and their resistance genes is necessary for early clinical diagnosis and appropriate treatment. This study combines recombinase polymerase amplification (RPA) and clustered regularly interspaced short palindromic repeats-association protein 13a (CRISPR-Cas13a) to establish a one-tube and two-step reaction systems for detecting the *mexX* gene in *P. aeruginosa*. The test times for one-tube and two-step RPA-Cas13a methods were 5 and 40 min (including a 30 min RPA amplification reaction), respectively. Both methods outperform Quantitative Real-time Polymerase Chain Reactions (qRT-PCR) and traditional PCR. The limit of detection (LoD) of *P. aeruginosa* genome in one-tube and two-step RPA-Cas13a is 10 aM and 1 aM, respectively. Meanwhile, the designed primers have a high specificity for *P. aeruginosa mexX* gene. These two methods were also verified with actual samples isolated from industrial settings and demonstrated great accuracy. Furthermore, the results of the two-step RPA-Cas13a assay could also be visualized using a commercial lateral flow dipstick with a LoD of 10 fM, which is a useful adjunt to the gold-standard qRT-PCR assay in field detection. Taken together, the procedure developed in this study using RPA and CRISPR-Cas13a provides a simple and fast way for detecting resistance genes.

## 1 Introduction

*Pseudomonas aeruginosa* is an opportunistic pathogen that is commonly found in hospitals. It can cause a variety of chronic infections, including urinary tract infections, bronchial dilation, and cystic fibrosis, all of which have a high recurrence rate and are difficult to treat (Gellatly and Hancock, 2013; Diggle and Whiteley, 2020; Shi et al., 2023). In addition, *P. aeruginosa* can cling to various surfaces of food processing equipment to form biofilms, endangering food safety and quality and potentially causing food-borne diseases (Teng et al., 2022; Ingrid et al., 2023; Silvia et al., 2023; Wu et al., 2023). Under the pressure of drug selection, *P. aeruginosa* continuously evolves and varies in its tolerance to a variety of commonly used antibiotics. Meanwhile, the drug-resistant mechanisms of *P. aeruginosa* are quite complex. However, excessive expression of efflux pumps’ bacterial active external discharge system is regarded to be one of the primary drug resistance mechanisms of *P. aeruginosa* (Eichenberger and Thaden, 2019). These efflux systems can pump out a wide range of molecules, including antibiotics with diverse structures and foreign biological substances, in order to reduce the concentration of intracellular antibacterial chemicals and prevent the development of multi-drug resistance in bacteria (Cunrath et al., 2019). There are at least 12 varieties of resistance nodulation division (RND) external pumps in *P.aeruginosa*, including MexAB-OprM, MexCD-OprJ, MexEF-OprN, MexXY-OprM, MexJK-OprM, MexGHI-OprM, MexMN-OprM, with MexAB-OprM, and MexXY-OprM being the most prevalent (Pesingi et al., 2019; Tsutsumi et al., 2019). In particular, MexXY over-expression is frequently found in the clinical isolates of drug-resistant *P. aeruginosa*, and it is becoming widely accepted that MexXY plays a substantial role in *P. aeruginosa* aminoglycoside resistance (Henríquez et al., 2020; Kei et al., 2021). A mutant missing MexXY showed increased resistance to aminoglycosides, erythromycin, and tetracycline but not to ß-lactams, chloramphenicol, or quinolones (Morita et al., 2001). Because of their significance in the drug tolerance mechanism, efflux pumps have emerged as a crucial therapeutic target. Antibiotic resistance can be combated by preventing these efflux pumps from working (Avakh et al., 2023). However, the modern quick and effective detection methods for efflux pump genes are scarce, limiting the prompt detection and treatment of resistant *P. aeruginosa*.

In prokaryotes, clustered regularly interspaced short palindromic repeats (CRISPR) and association protein (Cas) are immunological defensive mechanisms that resist the invasion of foreign genetic material (Manghwar et al., 2019). The nucleic acid enzyme domain in Cas13a has RNA endonuclease activity and the ability to cut RNA non-specifically. Cas13a can precisely identify and cut the target RNA in the case of the processed RNA molecule (crRNA). Cas13a randomly cuts adjacent RNA after its collateral-cutting capabilities is activated. This motivates Cas13a to create novel RNA-targeting tools and expand the application of CRISPR systems for gene detection (Abudayyeh et al., 2016; Zhao et al., 2022). By labeling RNA probes with fluorophores at both ends, CRISPR/Cas13a can detect RNA templates and amplify signals to achieve specific detection of target molecules (Abudayyeh et al., 2016; Kellner et al., 2019). More specifically, CRISPR-Cas13a-based detection techniques provide the following benefits and advantages: Cas13a has no protospacer adjacent motif (PAM) site limit, allowing it to target any sequences (Escalona-Noguero et al., 2021). Therefore, CRISPR-Cas13a has a promising application prospect in pathogenic testing.

Antibiotics overuse and misuse have been linked to the rapid emergence of drug-resistant bacteria (Tacconelli et al., 2018; Ranjbar et al., 2024). Bacterial resistance has grown to be an urgent issue that must be tackled. The Antimicrobial Susceptibility Test (AST) is a classic method for determining microbial drug resistance (Antimicrobial Resistance, AMR; Paul et al., 2022). Then physical, chemical, and molecular biology detection technologies emerged, including flow cytometry (Williams et al., 2017), Raman spectroscopy (Duan et al., 2016), and mass spectrometers (Lee et al., 2016), as well as immune hybridization (Shahid et al., 2021). For nucleic acid amplification, molecular biological detection technologies, such as Polymerase Chain Reaction (PCR; Niu et al., 2020), Quantitative Real-time PCR (qRT-PCR; Abramova et al., 2023), and whole-genome/metagenome sequencing (Boolchandani et al., 2019), are commonly used. Although most of the tactics outlined above are commonly utilized in practical settings, they are either ineffectual or necessitate complex apparatus. A developed isothermal nucleic acid amplification technique of Recombinase polymerase amplification (RPA) has significant advantages in terms of reaction time and equipment (Tan et al., 2022). Numerous researchers have imaginatively created novel nucleic acid detection technologies based on CRISPR-Cas13a and RPA (Gao et al., 2021; He et al., 2022; MacGregor et al., 2023; Miao et al., 2023). However, greater improvement of the sensitivity, precision, and experimental protocols of the RPA coupled Cas13a detection approach is required for visual molecular diagnostics.

Therefore, this study uses CRISPR/Cas13a with RPA technology to develop specific primers and probes for the identification of *mexX* in *P. aeruginosa*. The easy operation method with great sensitivity to the presence of *mexX* provides technical support for preventing and controlling *P. aeruginosa* resistance.

## 2 Materials and methods

### 2.1 Chemical reagents and materials

LwCas13a protein and its corresponding buffer were purchased from Magigen Biotech Co. Ltd. (Guangzhou, China). DNase I, T7 RNA polymerase, and recombinant RNase inhibitor were obtained from TaKaRa (Dalian, China). ChamQ Universal SYBR qPCR Master Mix was purchased from Vazyme (Guangzhou, China). Sangon Biotech (Shanghai, China) provided the NTP mixture (10 mM each), dNTP mixture (25 mM each), and Spin Column RNA Cleanup & Concentration Kit. DNA Constant Temperature Rapid Amplification Kit (Liquid Basic Type) and CRISPR Cas12/13 Hybridetect Test Note were purchased from Warbio Biotech (Nanjing, China).

### 2.2 Bacterial strains and nucleic acid preparation

In our laboratory, *P. aeruginosa* was isolated from spoiled wood paint lotion. Other pathogenic bacteria obtained from American Type Culture Collection (ATCC) and German Collection of Microorganisms and Cell Cultures included *Staphylococcus aureus* ATCC 6538P, *Escherichia coli* ATCC 8739*, Vibrio parahaemolyticus* ATCC 17802*, Salmonella enterica* ATCC 9115*, Klebsiella pneumoniae* ATCC 4352*, Lactobacillus paracasei* ATCC 334*, Bacillus paralicheniformis* ATCC 9945, *and Bacillus subtilis* DSM 23778. The nucleic acids of the above strains were extracted using the HiPure Bacterial DNA Kit (Magen Biotech Co., Ltd.; Guangzhou, China) according to the manufacturer’s instructions. The concentration of DNA was quantified using the Nanodrop One instrument (Thermo Fisher Scientific, Shanghai, China). Until they were used, all DNA samples were stored at-20°C.

### 2.3 Primer design for crRNA and RPA

The *mexX* gene sequences of *P. aeruginosa* were downloaded from the GenBank database of the National Center for Biotechnology Information (NCBI).^1^ After aligning gene sequences to identify conserved regions, 10 crRNAs were designed using the online Cas13 design tool.^2^ According to the selected target sequence design and relevant works of literatures (Liu et al., 2017; Gootenberg et al., 2018; Yang et al., 2023), a T7 promoter binding sequence was attached to the 5′-end of the designed crRNAs. Simultaneously, the PRIMER 5 program was used to design equivalent RPA primers between the upstream and downstream clips of the candidate target DNA. The RNA probe 1 is tagged with 6-FAM at the 5′ -end and uses BHQ1 at the 3′ -end (Zhou et al., 2020), whereas RNA probe 2 is designated with 6-FAM at the 5′ end and uses BIO at the 3′ end (Wang et al., 2023). The designed primers, probes, and oligonucleotides were synthesized by TsingkeBiotech (Beijing, China). All of the nucleic acid sequences used in this study are listed in Table 1.

**Table 1:** Sequence of primers, oligonucleotides and RNA probes in this study.

Name	Sequence (5′-3′)
*mexX*-crRNA 1	TAATACGACTCACTATAGGGGATTTAGACTACCCCAAAAACGAAGGGGACTAAAACATCGATCCGATCTACGTGAACTT
*mexX*-crRNA 2	TAATACGACTCACTATAGGGGATTTAGACTACCCCAA*AAACGAAGGGGACTAAAAC*GAAGTTCACGTAGATCGGATCGA
*mexX*-crRNA 3	TAATACGACTCACTATAGGGGATTTAGACTACCCCAA*AAACGAAGGGGACTAAAAC*GCGACACCCTTCACCTGGCCTTC
*mexX*-crRNA 4	TAATACGACTCACTATAGGGGATTTAGACTACCCCAAAAACGAAGGGGACTAAAACTCCACGTCTTCCACCACGCCCTG
*mexX*-crRNA 5	TAATACGACTCACTATAGGGGATTTAGACTACCCCAAAAACGAAGGGGACTAAAACCCACGTCTTCCACCACGCCCTGT
*mexX*-crRNA 6	TAATACGACTCACTATAGGGGATTTAGACTACCCCAAAAACGAAGGGGACTAAAACGATGATCCAGTCACGGCCCTGCA
*mexX*-crRNA 7	TAATACGACTCACTATAGGGGATTTAGACTACCCCAAAAACGAAGGGGACTAAAACCCAGCAGGAATAGGGCGACCAGG
*mexX*-crRNA 8	TAATACGACTCACTATAGGGGATTTAGACTACCCCAAAAACGAAGGGGACTAAAACCTGATGATCCAGTCACGGCCCTG
*mexX*-crRNA 9	TAATACGACTCACTATAGGGGATTTAGACTACCCCAAAAACGAAGGGGACTAAAACTGATGATCCAGTCACGGCCCTGC
*mexX*-crRNA 10 F	TAATACGACTCACTATAGGGGATTTAGACTACCCCAAAAACGAAGGGGACTAAAACATGATCCAGTCACGGCCCTGCAG
*mexX*-crRNA 1 R	AAGTTCACGTAGATCGGATCGATGTTTTAGTCCCCTTCGTTTTTGGGGTAGTCTAAATC
*mexX*-crRNA 2 R	TCGATCCGATCTACGTGAACTTCGTTTTAGTCCCCTTCGTTTTTGGGGTAGTCTAAATC
*mexX*-crRNA 3 R	GAAGGCCAGGTGAAGGGTGTCGCGTTTTAGTCCCCTTCGTTTTTGGGGTAGTCTAAATC
*mexX*-crRNA 4 R	CAGGGCGTGGTGGAAGACGTGGAGTTTTAGTCCCCTTCGTTTTTGGGGTAGTCTAAATC
*mexX*-crRNA 5 R	ACAGGGCGTGGTGGAAGACGTGGGTTTTAGTCCCCTTCGTTTTTGGGGTAGTCTAAATC
*mexX*-crRNA 6 R	TGCAGGGCCGTGACTGGATCATCGTTTTAGTCCCCTTCGTTTTTGGGGTAGTCTAAATC
*mexX*-crRNA 7 R	CCTGGTCGCCCTATTCCTGCTGGGTTTTAGTCCCCTTCGTTTTTGGGGTAGTCTAAATC
*mexX*-crRNA 8 R	CAGGGCCGTGACTGGATCATCAGGTTTTAGTCCCCTTCGTTTTTGGGGTAGTCTAAATC
*mexX*-crRNA 9 R	GCAGGGCCGTGACTGGATCATCAGTTTTAGTCCCCTTCGTTTTTGGGGTAGTCTAAATC
*mexX*-crRNA 10 R	CTGCAGGGCCGTGACTGGATCATGTTTTAGTCCCCTTCGTTTTTGGGGTAGTCTAAATC
*mexX*-RPA-F1	TAATACGACTCACTATAGGGAGCGAACGCGAGTACACCGAAGCGCAGAC
*mexX*-RPA-R1	ATGTCCTTGTCGGCGACACCCTTCACCTGG
*mexX*-RPA-F2	TAATACGACTCACTATAGGGCATCCAATGGACCGGCTCGCTGCGCGGGCT
*mexX*-RPA-R2	CTTCCAGGCGTCCGGGCAGCTCGCTGGTGATG
*mexX*-RPA-F3	TAATACGACTCACTATAGGGCTGATCCGTACCGCCCAGTCCGCCGTGGTC
*mexX*-RPA-R3	CGGCATGCTGGGCGGCGTTCTCGACGATC
mexX-qRT-PCR F	CCGTGCTGTTCCAGATC
mexX-qRT-PCR R	TCCTTGATCAGGTCGGCG
mexX-PCR F	GCGGAAGGTCAGGGTCAG
mexX-PCR R	GGTTTTCTGGGATTCCTCTTTG
RNA probe 1	6-FAM-UUGGCGUAAUCAUGGUCAUA-BHQ1
RNA probe 2	6-FAM-UUUUUUUUUUUUUUUUUUUU-BIO

F, forward primer; R, reverse primer. The T7 promoter is underlined; GGG is required for T7 transcription.

### 2.4 crRNA preparation

The templates for an oligonucleotide incorporating T7 promoters, repetitive sequences, and interval sequences have been developed to create the double strand DNA (dsDNA). Forward and reverse oligonucleotide DNA (5 μmol/L) were added to produce dsDNA templates for transcription. And each single-chain template was then mixed, annealed for 5 min at 95°C and naturally cooled to room temperature naturally. The crRNA was synthesized by incubating the above mixtures for 2 h at 42°C with T7 RNA polymerase (TaKaRa). To carry out the process, the synthesized crRNA was digested with DNase I (TaKaRa) at 37°C for 1 h to remove DNA templates, and then purified using the RNA Rapid Concentration and Purification kit (Sangon Biotech, Shanghai, China) in accordance with the manufacturer’s instructions. The crRNA concentration was then determined using the NanoDrop spectrophotometer (Thermo Fisher Scientific, Shanghai, China) and was subsequently kept at-80°C until use.

### 2.5 One-tube RPA-Cas13a

**Figure 1:**
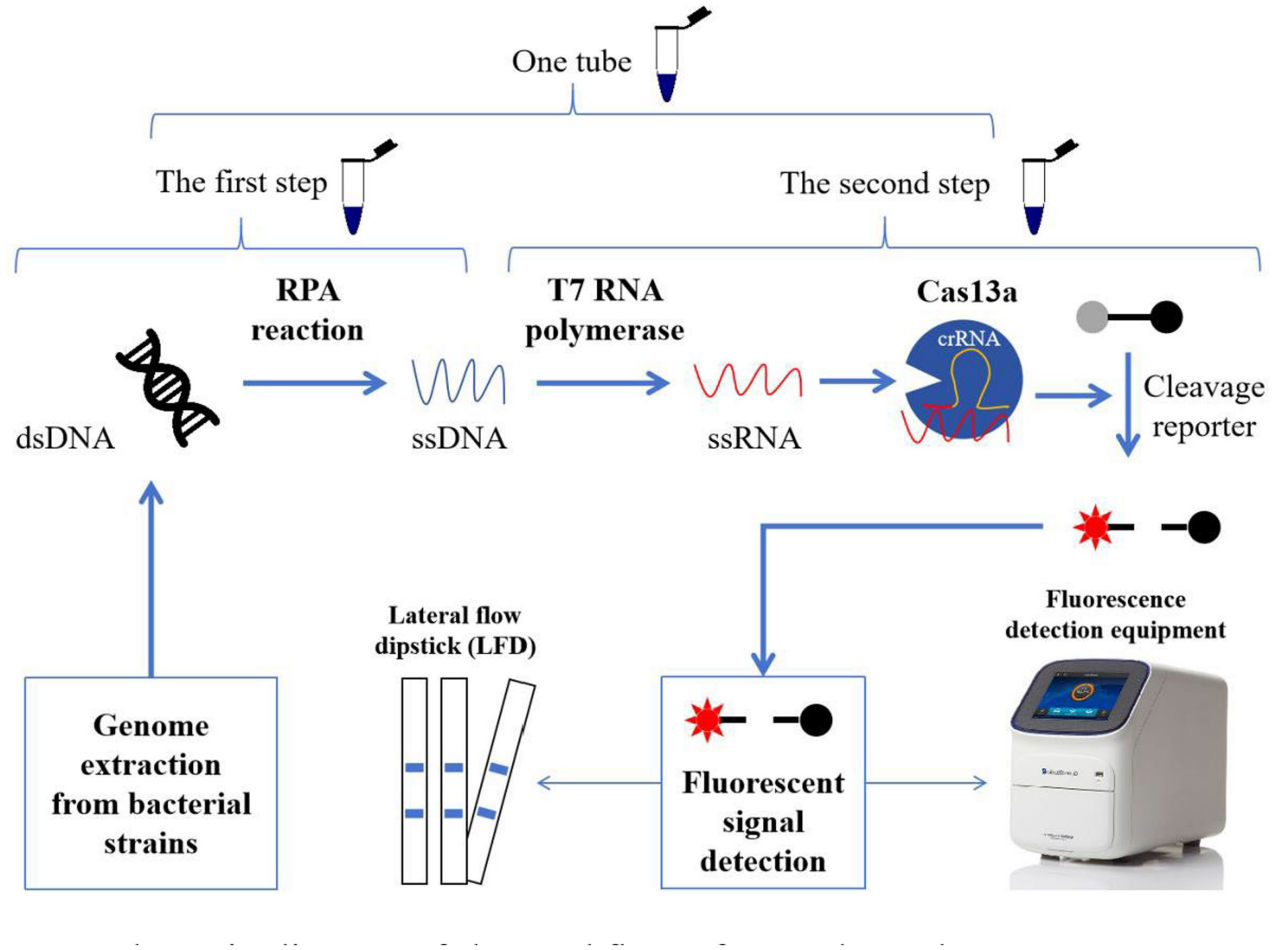
Schematic diagram of the workflow of one-tube and two-step RPA-Cas13a detection methods. In the two-step process, the extracted genomes from bacterial strains were introduced to the RPA reaction to amplify the targeted sequences (ssDNA). And then, the acquired ssDNAs were translated into ssRNA using T7 RNA polymerase. Cas13a activity was initiated when the target sequences in ssRNA were identified by crRNA. Meanwhile, the active Cas13a exhibits the auxiliary cleavage activity to cleave the RNA fluorescence probe, which could be detected by fluorescence detection equipment (fluorescence quantitative PCR instrument or enzyme-linked immunosorbent assay instrument) or LFD. If the first and second steps were performed in one tube, we referred to this as one-tube RPA-Cas13a detection method.

For the one-tube RPA-Cas13a assay, RPA amplification and CRISPR-Cas13a were coupled in a one-tube reaction system (Figure 1). Briefly, the 50 μL one-tube reaction system consisted of 18 μL C buffer (Warbio Biotech Co. Ltd.; Guangzhou, China), 5 μL L buffer (Warbio), 12 μL P-core (Warbio), 1 μL each of 10 μM forward and reverse primers, 1.5 μL 10 mM dNTPs, 1 μL target DNA template, 2 μL 25 mM NTP mix, 1 μL recombinant RNase inhibitor, 1 μL 10 μM RNA-probe, 1 μL T7 RNA polymerase, 0.4 μL 2 μM Cas13a, 1 μL 12 nM crRNA, and added RNase-free ddH_2_O to 50 μL. After gently spinning the reaction mixtures, 2.5 μL of 280 mM magnesium acetate (MgOAc) was added. Finally, the reactions were then run at 39°C for 20 min, and the fluorescence signal was collected every 1 min on a QuantStudio™ Real-Time PCR Software (Applied Biosystems, Waltham, MA, United States) with three replicates set for each sample.

### 2.6 Two-step RPA-Cas13a

In the two-step RPA-Cas13a assay, the crucial RPA reactions were first conducted according to the instructions of the DNA constant temperature rapid amplification kit (Warbio, China; Figure 1). Each 50 μL RPA reaction volume contained the following elements: 20 μL C buffer, 5 μL L buffer, 12 μL P-core, 2 μL each of 10 μM forward and reversed primers, 1.5 μL 10 mM dNTPs, 1 μL target DNA template, and 4 μL RNase-free ddH_2_O. The reaction mixtures were gently vortexed and spun. Then, 2.5 μL of 280 mM MgOAc was added and thoroughly mixed to start the reaction, which would last for 30 min at 39°C. Subsequently, a 50 μL CRISPR-Cas13a reaction system was performed with 5 μL 10 × reaction buffer, 2 μL 25 mM NTP mix, 0.8 μL recombinant RNase inhibitor, 1 μL PA-crRNA, 1 μL 10 μM RNA-probe, 0.6 μL T7 RNA polymerase, 0.4 μL 2 μM Cas13a, 4 μL RPA products and added RNase-free ddH_2_O to 50 μL. Three replicates were set up for each sample, and the reactions were carried out at 39°C for 20 min after mixing. The fluorescence signal was collected every minute using the QuantStudio^™^ Real-Time PCR program.

### 2.7 LwCas13a feasibility analysis for nucleic detection

The crRNA 1 with the highest predicted score in online website design was selected for the detection system to confirm the activity of the LwCas13a protein and examine if a detection system could be successfully established. In the first, second, and third groups, equivalent amounts of RNase-free H_2_O were introduced to the reaction system in place of crRNA 1, DNA, and LwCas13a, which served as negative controls. Instead of DNA, crRNA 1, and LwCas13a protein, the blank control group received an equal amount of RNase-free H_2_O. After mixing, the reactions were run for 20 min at 39°C, and the fluorescence signal was collected every 1 min on a QuantStudio^™^ Real-Time PCR Software with three replicates.

### 2.8 Target crRNA screening

To ensure the excellent activity of the Cas13a protein, 10 crRNAs were obtained via the online Cas13 design tool. And three pairs of RPA isothermal amplification primers were designed based on the conserved sequence to match the crRNA. Using the same batch of extracted DNA products, the CRISPR-Cas13a system, which contains 10 distinct crRNAs, was next tested for detection efficiency. The endpoint fluorescence value was recorded for comparison.

### 2.9 Reaction condition optimization

The Cas13a-crRNA-fluorescent measurement signal was used to optimize the detection system, and the fluorescence value was simultaneously selected for comparison. The samples for the optimization procedure are *P. aeruginosa* DNA samples with the same initial concentration. A single variable should be controlled to optimize the following parameters: reaction temperatures (37, 38, 39, 40, and 41°C), primer concentrations (0.6–1 μM), LwCas13a concentrations (0–40 nM), and crRNA concentrations (0–48 nM).

### 2.10 Sensitivity and specificity of one-tube and two-step RPA-Cas13a

To compare the minimal LoD of these two approaches, the extracted *P. aeruginosa* nucleic acids are employed as a template to dilute a series of gradients 10 times, resulting in distinct concentration standards. The gradient diluted dsDNA standards were detected using the one-tube and two-step RPA-Cas13a methods. We used qRT-PCR as the reference test, which was conducted according to the guidelines provided in the following section. The specificity of the one-tube and two-step RPA-Cas13a methods was tested by using the genomes extracted from *P. aeruginosa, S. aureus* ATCC 6538P*, E. coli* ATCC 8739*, V. parahaemolyticus* ATCC 17802*, S. enterica* ATCC 9115*, K. pneumoniae* ATCC 4352*, L. paracasei* ATCC 334*, B. paracaeniformis* ATCC 9945, and *B. subtilis* DSM 23778.

### 2.11 Verification of RPA-Cas13a using industrial products

In our laboratory, 38 *P. aeruginosa* were previously isolated from industrial products (spoiled daily chemical products and coatings; Supplementary Table 1). To validate the usefulness and practicality of the RPA-Cas13a assay, nucleic acids from these real specimens were extracted and detected using the one-tube and two-step RPA-Cas13a assay methods mentioned above. Meanwhile, qRT-PCR and PCR were also employed to detect these samples, and the results were finally compared with those obtained using RPA-Cas13a.

### 2.12 qRT-PCR

After a 10-fold gradient dilution, the DNA template was detected using a fluorescence quantitative PCR kit (TaKaRa). The reaction mixture contains 10 μL 2 × ChamQ Universal SYBR qPCR Master Mix, 0.4 μL of each primer pair at 10 μM, 1 μL template DNA, and ddH_2_O to 20.0 μL. qRT-PCR was performed on the QuantStudio^™^ Real-Time PCR software instrument, and the reaction was run as follows: 95°C for 30 s, followed by 40 cycles of 95°C for 10 s, and then 60°C for 30s.

### 2.13 PCR detection

For comparison with RPA-Cas13a, the traditional PCR was also used to amplify the targeted *mexX* gene from the strains used in this study. The following steps were taken to prepare the PCR reaction system: 5 μL 2 × Rapid Taq Master Mix (Vazyme, Nanjing, China), 0.2 μL of each primer (10 μmol/L), 4.4 μL ddH_2_O, and 0.2 μL template DNA. The mixtures were subsequently subjected to 30 cycles of PCR using a PCR instrument (T100 Thermal Cycler; Bio-Rad Laboratories) with the following reaction parameters: 95°C for 5 min, 95°C for 15 s, annealing at 57°C for 15 s, and 30 s extending at 72°C. One more extension cycle at 72°C for 5 min was needed to complete the reaction.

### 2.14 Establishment of Cas13a lateral flow dipstick

The practical workflow for this test consists of three steps. First, the gene was amplified using RPA and translated to ssRNA. Then, this nuclease recognized crRNA and cut off the reporter molecule using its collateral cutting capability. Lastly, lateral flow dipstick assays were performed using the identical reaction components as the fluorescent detection assays, but using 400 nM FAM-20 U-Biotin instead of the fluorescent reporter. And the reaction was incubated for 30 min at 39°C. A lateral flow dipstick (Warbio, Nanjing, China) was then inserted into the tube and incubated at room temperature for 5 min, during which the result was recorded. It was noteworthy that, whereas negative samples should only have a C line, positive samples should display two lines (T and C lines).

### 2.15 Statistical analysis

The Cas13a-crRNA-fluorescence test signals were recorded and expressed as the mean of at least three independent reactions plus standard deviation (SD). GraphPad Prism 9.0 was used to do the analysis of variance and create the figures.

## 3 Results

### 3.1 Feasibility analysis of LwCas13a for nucleic detection

**Figure 2:**
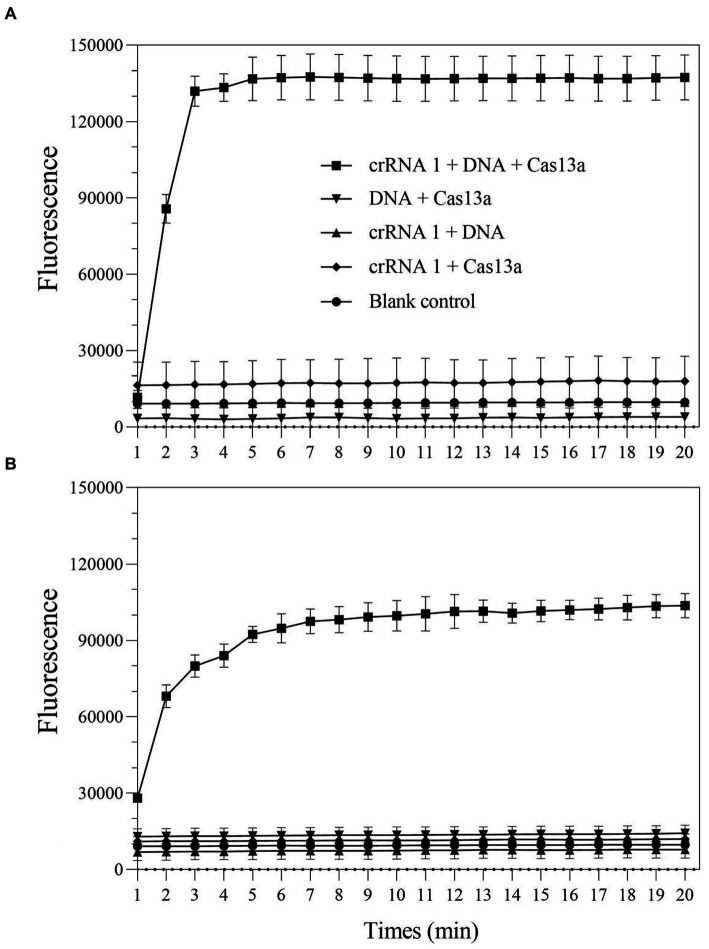
The collateral-cutting activity of CRISPR-Cas13a. **(A)** one-tube RPA-Cas13a detection; **(B)** two-step RPA-Cas13a detection.

To verify whether the LwCas13a protein exhibited the expected ribonuclease activity, a comprehensive Cas13a reaction system is developed using positive genes as the detection template. Additionally, in order to quantify fluorescence kinetics, negative and blank control groups were set up. The results demonstrated that when any of the following elements were not present in the detection system: LwCas13a, crRNA 1, or DNA/RPA product, the fluorescence signal remained essentially unchanged (Figure 2). When LwCas13a, crRNA 1, and DNA/RPA products were all present at the same time, the fluorescence signal increased noticeably, indicating that the Cas13a protein was performing its collateral cleavage activity in this circumstance (Figure 2). The fluorescence signal developed quickly over time and it only took 5 min for a single tube to react (Figure 2A), whereas the two-step method took roughly 10 min (Figure 2B). Following that, whether utilizing a single tube or a two-step procedure, the fluorescence kinetic curves approached a plateaued stage (Figure 2). These results demonstrated that the LwCas13a protein can precisely recognize target RNA and exhibits the anticipated auxiliary cleavage activity to cleave the RNA fluorescence probe in the system, resulting in a signal with high fluorescence intensity. The one-tube and two-step Cas13a fluorescence detection systems based on RPA and Cas13a have been effectively constructed.

### 3.2 Different crRNAs exhibit varying *mexX* detection efficiencies

**Figure 3:**
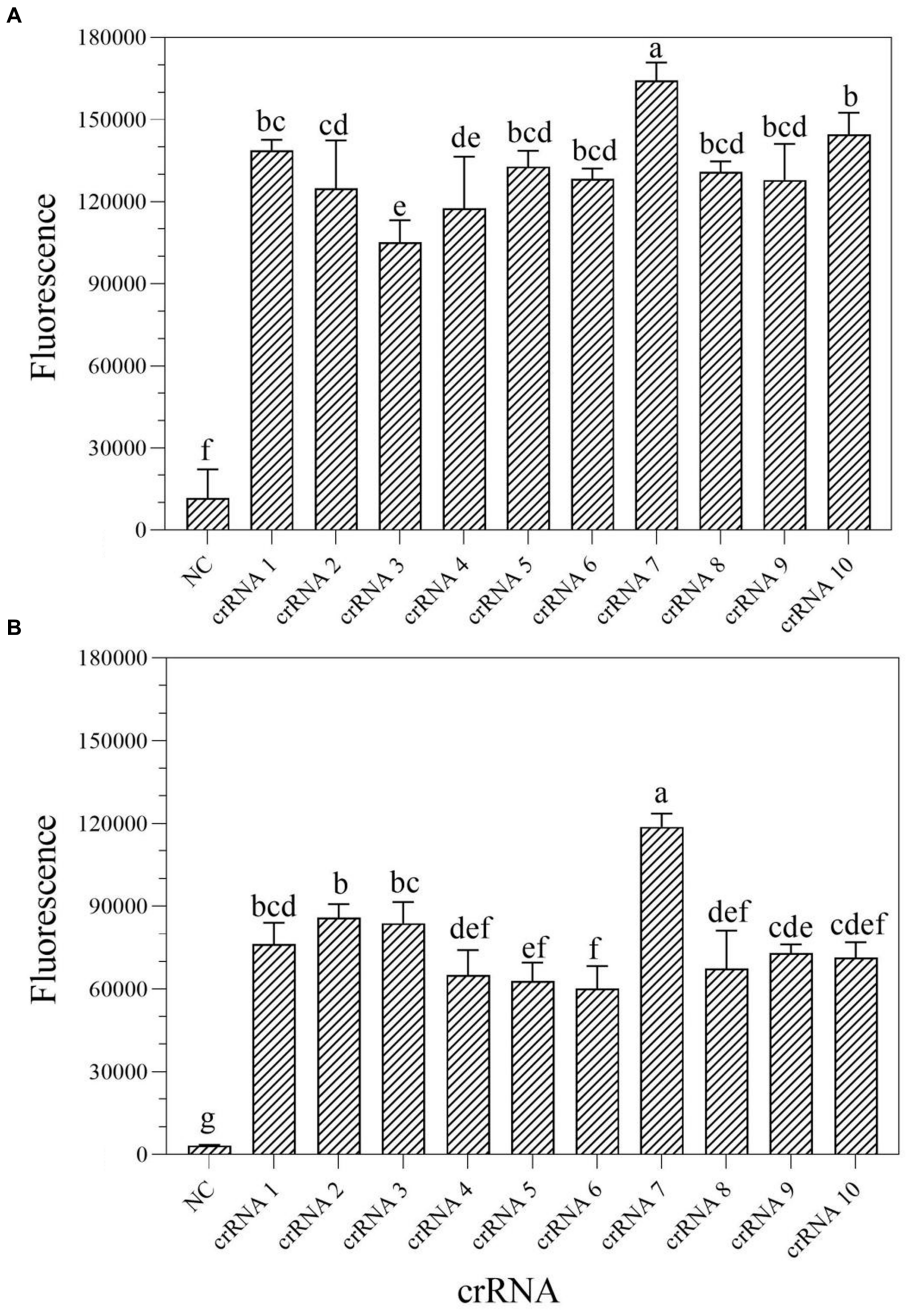
Screen of crRNAs for one-tube **(A)** and two-step **(B)** RPA-Cas13a detection systems. When Cas13a reacted with different crRNAs, all of the fluorescence signals were obtained at 10 min. All assays were performed in triplicate. Mean values and standard deviations are shown. Values with different letters are statistically different from each other for a given gene according to Fisher’s LSD test (*p* < 0.05). NC, negative control.

According to the earlier researches, crRNA is the primary factor affecting Cas13a cleavage activity (Liu et al., 2017; Yang et al., 2023). To improve the cleavage activity of LwCas13a, 10 crRNAs targeting the *mexX* gene were designed based on the Cas13a protospacer flanking site (PFS) motif. To identify the optimal crRNA for further research, the one-tube and two-step RPA-Cas13a fluorescence assays were performed. The fluorescence signals were recorded at 1 min intervals using a QuantStudio™ Real-Time PCR. The results demonstrated that the activity of the 10 different crRNA candidates is much higher than the no-target control (Figure 3). Within 10 min, all crRNAs produced fluorescence signals. However, crRNA 7 generated the maximum fluorescence intensity in both one-tube (Figure 3A) and two-step (Figure 3B) RPA-Cas13a detection systems, implying that crRNA 7 had enhanced activity for target DNA detection. Therefore, crRNA 7 was chosen as the optimal crRNA for the subsequent one-tube and two-step RPA-Cas13a detection optimizations.

### 3.3 RPA-Cas13a detection system can be affected by different parameters

**Figure 4:**
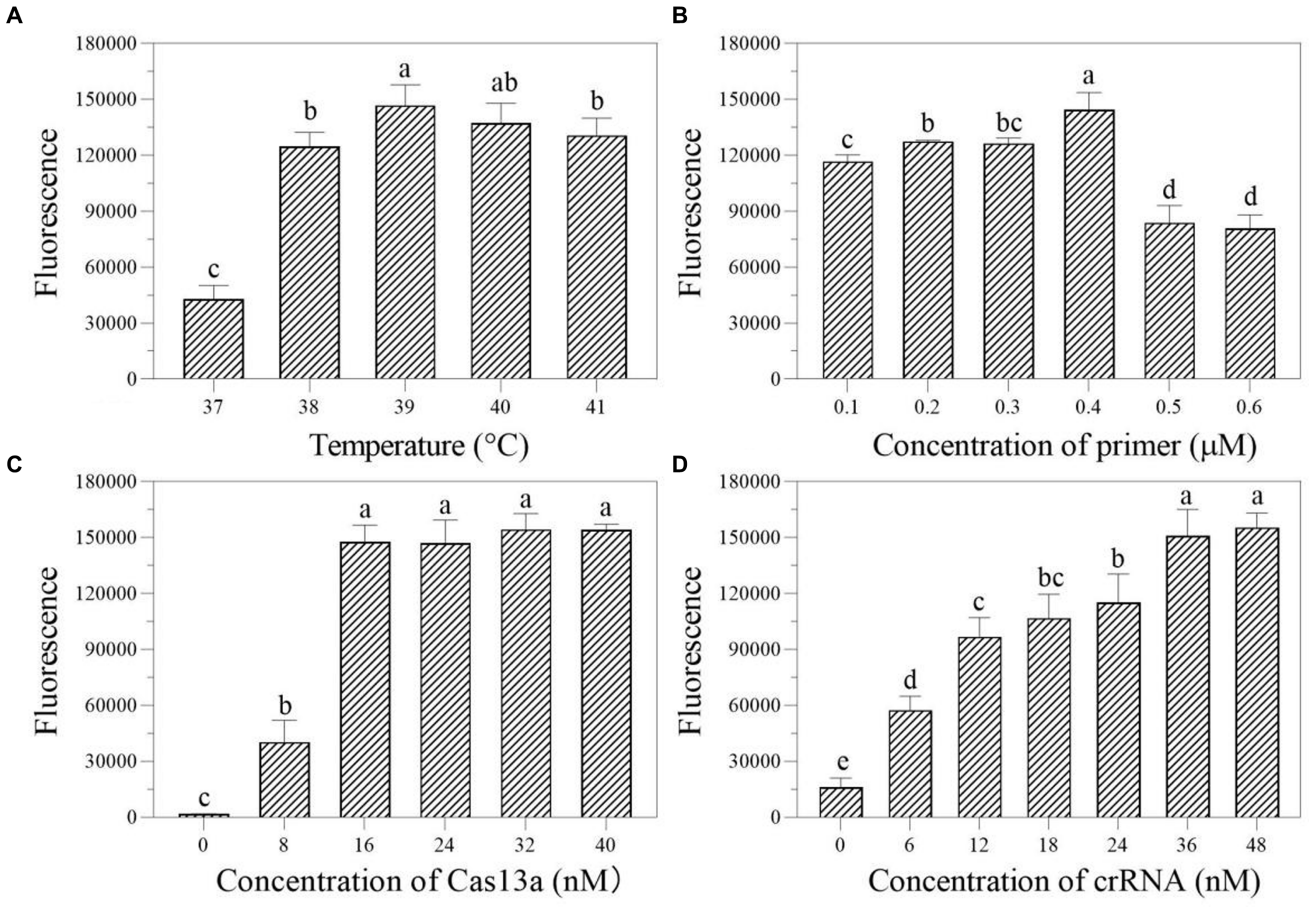
The effect of temperature (**A**; °C), primer concentration (**B**; μM), Cas13a concentration (**C**; nM), and crRNA concentration (**D**; nM) on the fluorescence in one-tube RPA-Cas13a detection system. All assays were performed in triplicate. Mean values and standard deviations are provided. Values with different letters are statistically different from each other for a given gene according to Fisher’s LSD test (*p* < 0.05).

In the one-tube RPA-Cas13a detection system, we first evaluated the effect of temperatures (37, 38, 39, 40, and 41°C) on fluorescence excitation of probes and discovered that 39°C exhibited the greatest fluorescence signals (Figure 4A). Then, the best fluorescence signal was obtained at an RPA primer concentration of 0.4 μM at 39°C conditions (Figure 4B). At 39°C and 0.4 μM primers, the optimum LwCas13a concentration was then determined. The results demonstrated that raising the LwCas13a concentration increased the fluorescence signal from 0 to 16 nM. There was only a minor fluorescence enhancement at LwCas13a concentrations greater than 16 nM (Figure 4C). Finally, we investigated the ideal crRNA concentration at 39°C, 0.4 μM primer, and 16 nM LwCas13a. Despite using less crRNA, the fluorescence generated is equivalent to 48 nM. Thus, the optimal crRNA concentration was determined to be 36 nM (Figure 4D). As a result, for one-tube, the ideal RPA-Cas13a conditions were 39°C, 0.40 μM primer, 16 nM LwCas13, and 36 nM crRNA concentration.

**Figure 5:**
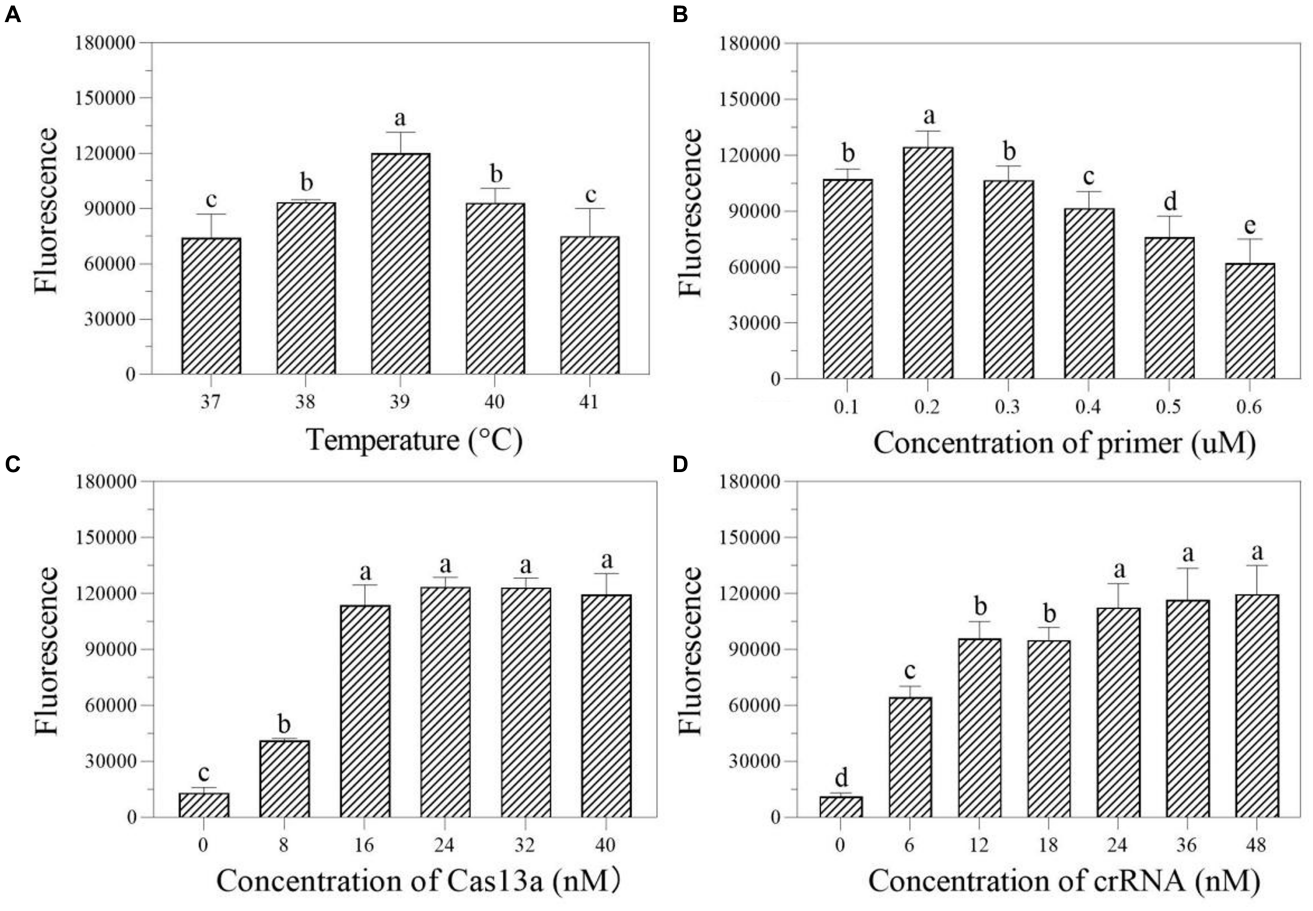
The effect of temperature (**A**; °C), primer concentration (**B**; μM), Cas13a concentration (**C**; nM), and crRNA concentration (**D**; nM) on the fluorescence in two-step RPA-Cas13a detection system. All assays were performed in triplicate. Mean values and standard deviations are shown. Values with different letters are statistically different from each other for a given gene according to Fisher’s LSD test (*p* < 0.05).

We first investigated the ideal reaction temperature for the two-step RPA-Cas13a reaction using a 0.40 μM primer concentration. The fluorescent signal reaches its maximum at 39°C, which was considered as the optimal temperature (Figure 5A). At 39°C, the ideal primer concentration was 0.2 μM (Figure 5B). Meanwhile, the fluorescence signal was enhanced at LwCas13a concentration between 0 and 16 nM, and this value hardly changed when the Cas13a concentration was over 16 nM (Figure 5C). Finally, at 39°C, 0.2 μM primer concentration, and 16 nM LwCas13a concentration, we explored the optimal crRNA concentration. It generated fluorescence comparable to the concentration range of 24–48 nM (Figure 5D). Thus, we determined that 24 nM was the optimal crRNA concentration (Figure 5D). Consequently, the optimal parameters for the two-step RPA-Cas13a were 39°C, 0.2 μM primer concentration, 16 nM LwCas13a, and 24 nM crRNA.

### 3.4 Sensitivity of one-tube and two-step RPA-Cas13a detection systems

**Figure 6:**
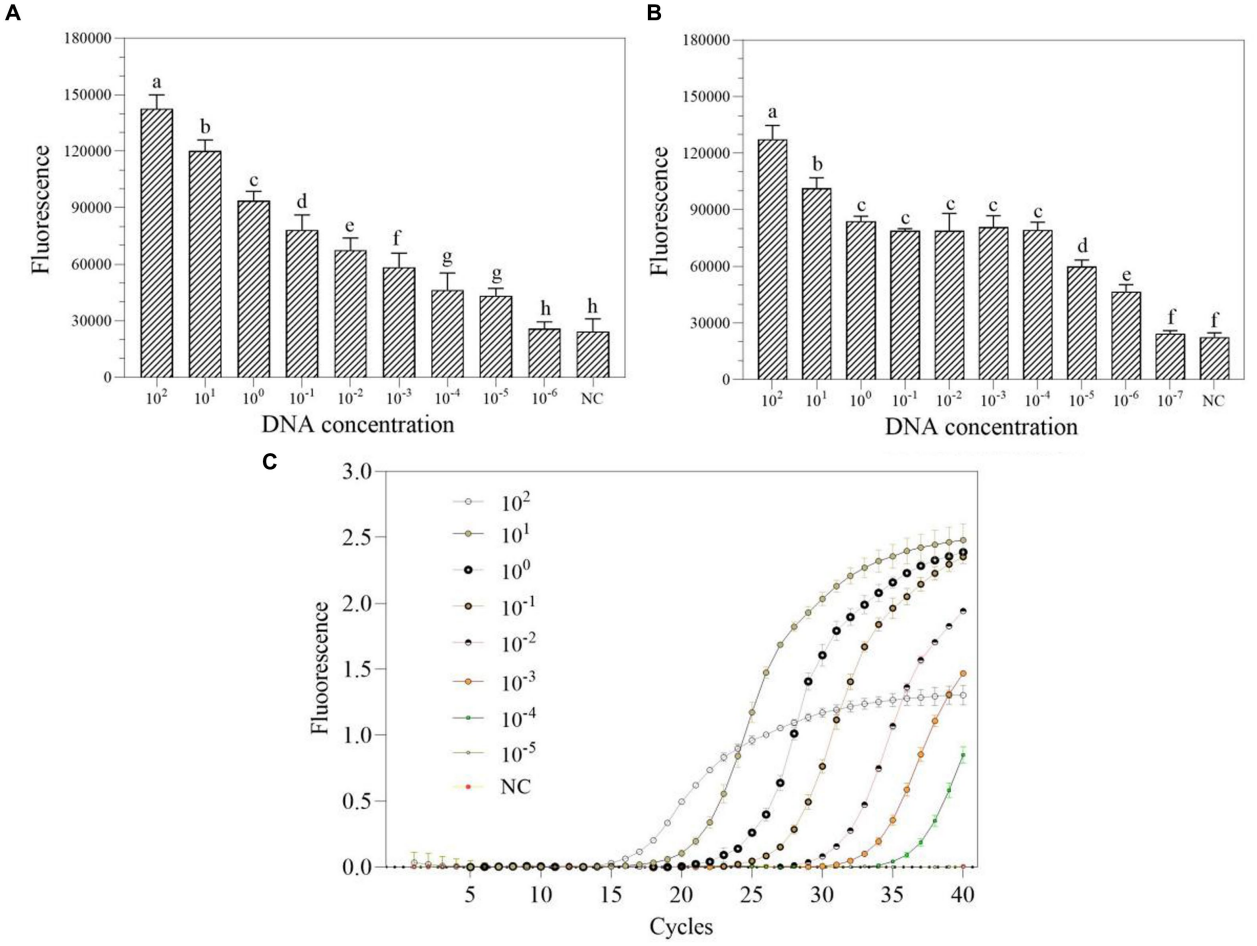
The sensitivity of one-tube **(A)** and two-step **(B)** RPA-Cas13a detection systems as well as real-time PCR **(C)**. 10^2^–10^−7^ (pM) represented the concentration of *P. aeruginosa* DNA standards. All assays were performed in triplicate. Mean values and standard deviations are shown. Values with different letters are statistically different from each other for a given gene according to Fisher’s LSD test (*p* < 0.05). NC, negative control.

The DNA at a starting concentration of 100 pM was diluted 10 times with RNase-free H_2_O to generate the detection concentration gradient. The sensitivity of one-tube and two-step RPA-Cas13a for *P. aeruginosa* was assessed using this gradient. The results demonstrated that the LoD for one-tube RPA-Cas13a was 10 aM (Figure 6A), slightly less than the LoD for two-step RPA-Cas13a (1 aM; Figure 6B). However, qRT-PCR exhibited a higher LoD of 100 aM (Figure 6C).

### 3.5 Specificity of one-tube and two-step RPA-Cas13a detection systems

**Figure 7:**
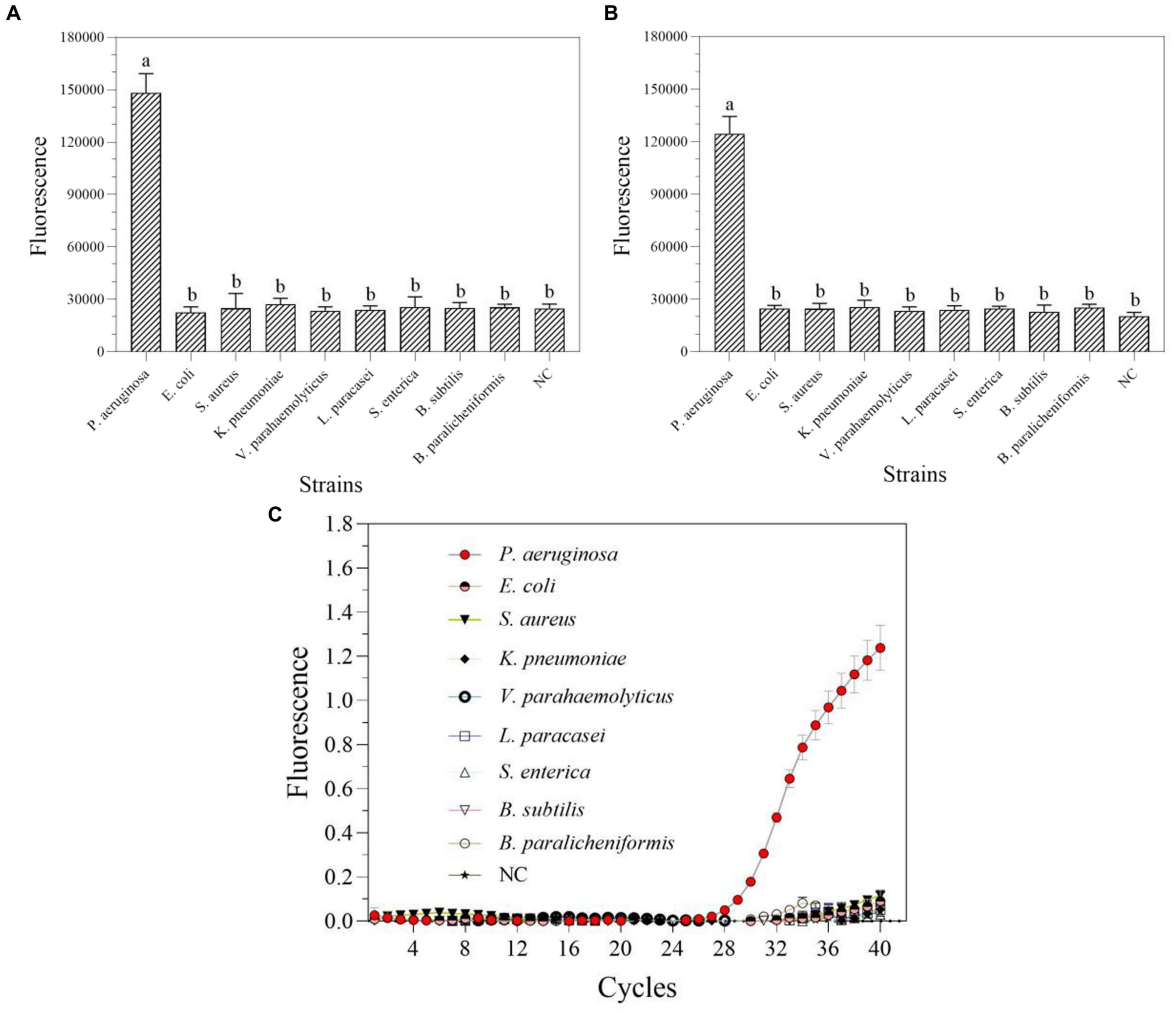
The specificity of one-tube **(A)** and two-step **(B)** RPA-Cas13a detection systems as well as real-time PCR **(C)**. All assays were performed in triplicate. Mean values and standard deviations are shown. Values with different letters are statistically different from each other for a given gene according to Fisher’s LSD test (*p* < 0.05). NC, negative control.

In nucleic acid testing, specificity is a fundamental issue: a suitable level of specificity improves detection accuracy by lowering false positive results and inaccurate conclusions (Antropov and Stepanov, 2023). Under optimal conditions, a particular experimental investigation was carried out to assay the degree of specificity of the RPA-Cas13a nucleic acid detection method. Only *P. aeruginosa* displayed detection signals among the nine common bacteria for specificity assessment without cross-reactions whether using one-tube (Figure 7A) or two-step RPA-Cas13a (Figure 7B). A comparable degree of specificity was also shown by qRT-PCR (Figure 7C). Thus, the target gene of *mexX* can be precisely identified using one-tube or two-step CRISPR/Cas13a-based detection method, which exhibits the same specificity as qRT-PCR.

### 3.6 Actual sample detection performance using RPA-Cas13a

**Figure 8:**
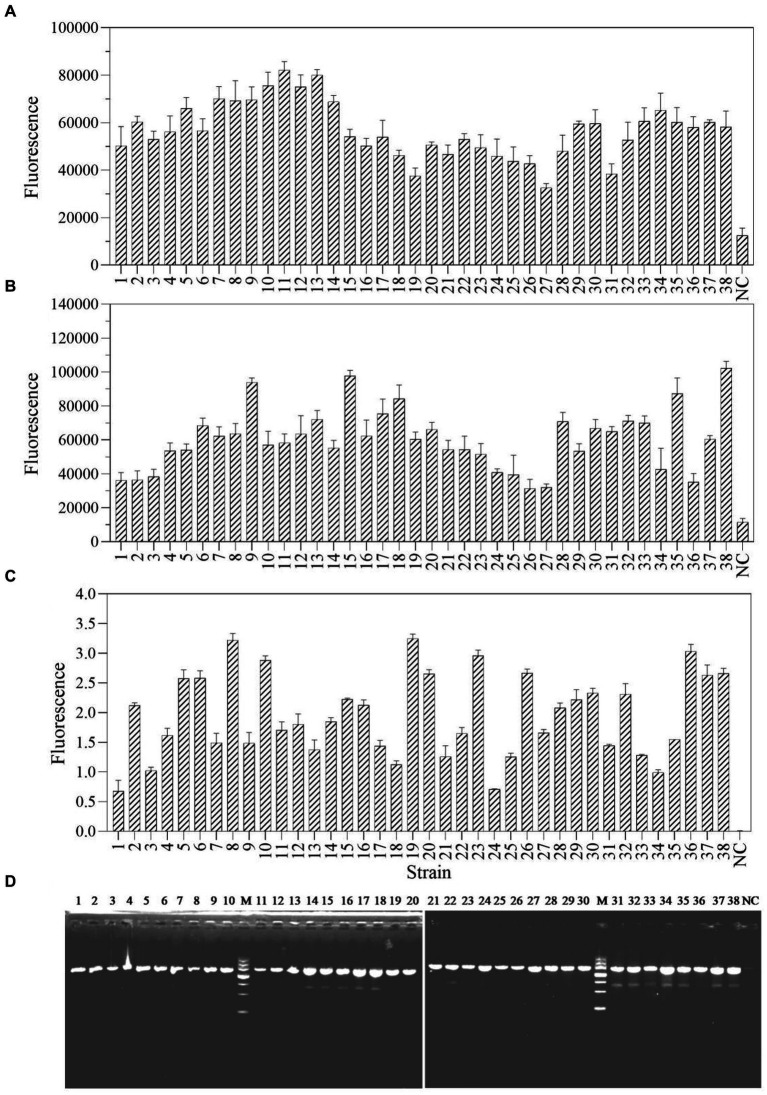
The sample consistency of four different detection methods: one-tube RPA-Cas13a **(A)**, two-step RPA-Cas13a **(B)**, qRT-PCR **(C)** at the 40 cycles, and traditional PCR **(D)**. M: DNA marker III, Tiangen Biotech Co. Ltd., China (from bottom to top: 200 bp, 500 bp, 800 bp, 1,200 bp, 2,000 bp, 3,000 bp, and 4,500 bp). NC, negative control.

The one-tube and two-step RPA-Cas13a methods were used to select and detect a total of 38 actual samples from various industrial products in order to assess the applicability of these two detection methods for *P. aeruginosa* in real samples. The results demonstrated that 38 samples, using both one-tube (Figure 8A) and two-step RPA-Cas13a (Figure 8B), tested positive for the target gene of *mexX*. Additionally, qRT-PCR (Figure 8C) and traditional PCR (Figure 8D) also demonstrated the same efficiency. We conclude that the one-tube and two-step procedures exhibit high agreement with qRT-PCR and traditional PCR, suggesting that these two RPA-Cas13a detection methods could be used to real samples.

### 3.7 Establishment of RPA-Cas13a-LFD

**Figure 9:**
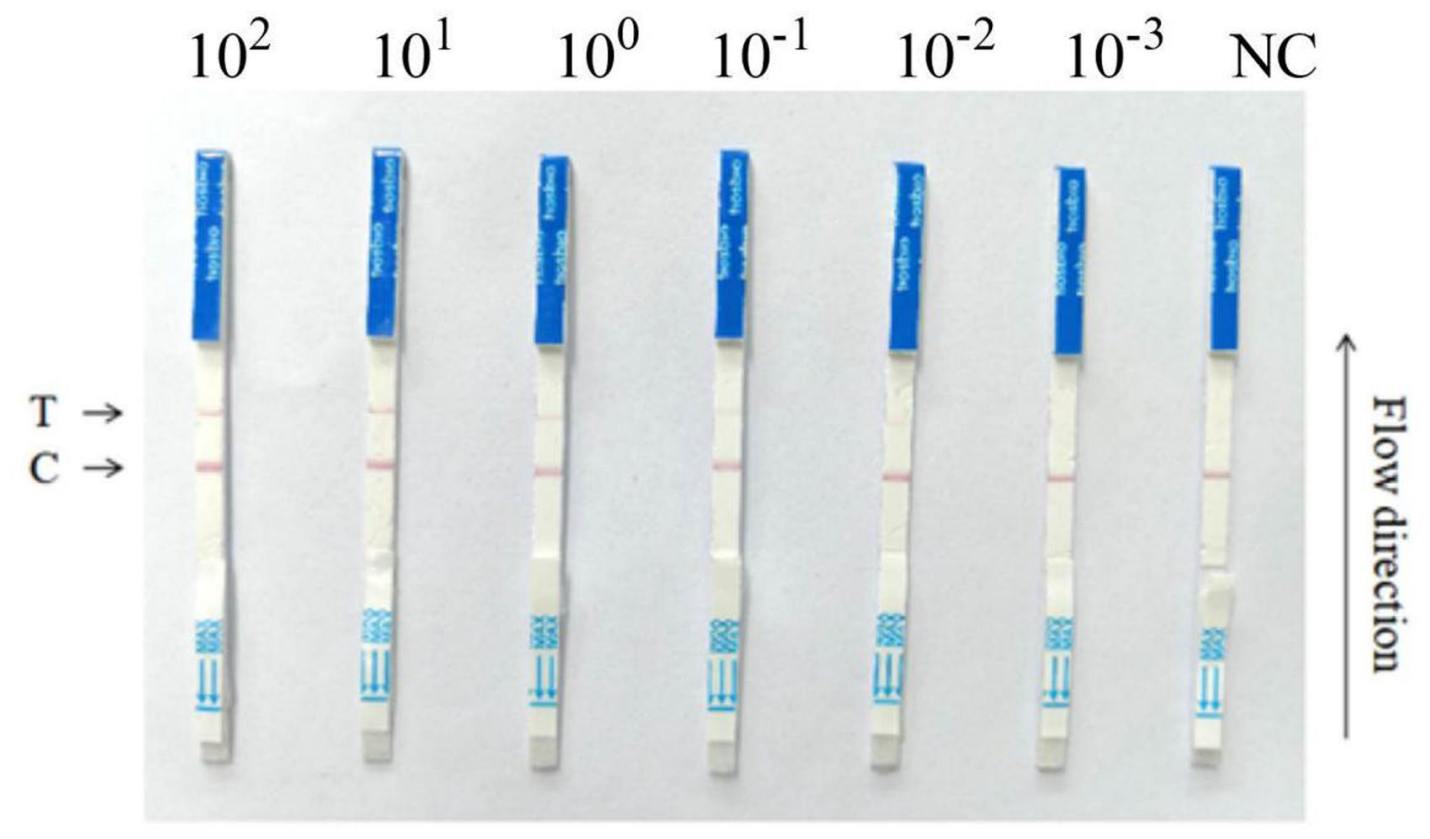
RPA-Cas13a-LFD detection of the *P. aeruginosa* DNA at various concentrations. 10-fold serial dilution of target DNA was used as the detection template. A RPA-Cas13a-LFD assay for on-site detection was developed using RPA, LwCas13a, and LFD. A biotin-tagged FAM-RNA reporter was used to create the LFD. For the negative control, an anti-FAM antibody-gold nanoparticle was conjugated to the FAM-RNA-biotin reporter, and the conjugation was intercepted by a biotin ligand at the control line. For the positive control, the FAM-RNA-biotin reporter was cleaved, and the anti-FAM antibody-gold nanoparticle conjugate accumulated at the test line. The two-step RPA-Cas13a-LFD detection reactions were performed using the following LoDs: 100 pM, 10 pM, 1 pM, 100 fM, 10 fM, 1 fM; NC, negative control; T, test line; C, control line.

To assess if the RPA-Cas13a detection method established in this study can directly apply the commercialized LFD to detect its results, a more practical and visually appealing detection strip method called RPA-Cas13a-LFD was developed. The results indicated that the initial template concentration affected the appearance of two lines and the LoD of the two-step RPA-Cas13a was 10 fM (Figure 9). The investigation demonstrated that commercial flow strips can be used to identify diagnostic results.

## 4 Discussion

Rapid, low-cost, and sensitive nucleic acid detection could help with pathogen identification, genotyping analysis, and disease monitoring at the point of care. Since the CRISPR-Cas13a-based molecular detection platform termed SHERLOCK was established with RPA in 2017 (Gootenberg et al., 2017), many research groups have attempted to optimize, improve, and extend this system. Up to now, this platform has been used to detect a number of viruses, such as syndrome coronavirus 2 (SARS-CoV-2; Cao et al., 2022; López-Valls et al., 2022; Su et al., 2022) and coronavirus disease (COVID-19). Meanwhile, CRISPR-Cas13a was also used to identify a number of bacterial strains, including *Aspergillus fumigatus* (Li et al., 2022), *Staphylococcus aureus* (Zhou et al., 2020), *Salmonella* spp. (An et al., 2021), *Neisseria gonorrhoeae* (Allan-Blitz et al., 2023) and *Group B Streptococci* (GBS; Li Z. et al., 2023). Moreover, the CRISPR-Cas13a technology was also utilized to identify multiple genes, such as toxin genes in *Clostridioides difficile* (Jiang et al., 2023), the *mecA* and *clfA* genes in *methicillin-resistant S. aureus* (MRSA; Liu et al., 2023), the *blaKPC* gene in *Enterobacterales* (Liang et al., 2023), the *lcrV* gene in *Yersinia pestis* (Schultzhaus et al., 2021). Similarly, in this study, we established a one-tube and two-step RPA-Cas13a platform (Figure 1) to detect the *mexX* gene from *P. aeruginosa*, with respectable sensitivity and reasonable specificity.

Several factors can influence the efficacy of one-tube and two-step RPA-Cas13a detection methods, including the reagent buffer used and the sequence of the RNA reporter (Gootenberg et al., 2018), as well as the concentration of Cas13a and used crRNA, reaction temperature, and RNA report probe concentration (Hu et al., 2022; Huang et al., 2022). The results of this study show that while the ideal crRNA, reaction temperature, and Cas13a concentrations of one-tube and two-step RPA-Cas13a techniques remain consistent, the optimal primer and crRNA concentrations varied somewhat between these two methods (Figures 3–5). Similar results were also discovered in prior references, which indicated that the optimal temperature and primer concentration for the one-tube and two-step methods are the same (An et al., 2021). The fundamental cause of the differences in optimum reaction conditions between this study and previous findings is the concentration of individual reagents in the systems, such as RNase inhibitors and total buffer. More diluted primer concentrations were found to improve both raw signal and quantitative accuracy in two methods, indicating that the reaction does not saturate at lower primer concentrations (Gootenberg et al., 2018). When it is insufficient, many Cas enzymes are unable to bind to crRNA to form complexes, leaving only a small number of active Cas enzymes, reducing the reaction’s efficiency (Hu et al., 2022). The Cas13a nuclease was fully activated throughout the system since the Cas13a concentration selected following the optimization of the two methods was the same (16 nM; Figures 4, 5), indicating that this concentration is sufficient to cut the RNA probe.

In this study, the LoD of the RPA-Cas13a platform can reach the fM or even aM level (Figure 6), which is comparable to or greater than previous studies. For example, the LoDs for hemagglutinin (HA) and neuraminidase (NA) genes of the H7N9 virus are 1 fM (Liu et al., 2019). Max J. Kellner detected synthetic DNA with a LoD of 20 aM (Kellner et al., 2019). In this study, one-tube and two-step RPA-Cas13a detection methods can detect DNA targets at concentrations as low as 10 aM (Figure 6A) and 1 aM (Figure 6B), respectively. Obviously, the sensitivity of the two-step RPA-Cas13a method is higher than that of the one-tube method in these two methods, which is consistent with the discoveries of An et al. (2021) and Arizti-Sanz et al. (2020).

The one-step method reduces nucleic acid detection sensitivity, owing to certain components of the CRISPR reagent buffer that limit nucleic acid amplification (Arizti-Sanz et al., 2020). For instance, magnesium ions in the CRISPR-Cas buffer may have an effect on the RPA reaction, reducing or stopping the RPA reaction’s ability to replicate. Furthermore, the Cas13a enzyme can identify and specifically cleave sample nucleic acid during the RPA amplification in conjunction with Cas13a detection, reducing the efficiency of the nucleic acid amplification and thus the sensitivity and detection efficiency (Wang et al., 2019). Moreover, the T7 RNA polymerase in the CRISPR-Cas13a reagent can impair the efficiency of the RPA-amplified dsDNA to ssRNA, hence hindering the nucleic acid amplification step. They fundamentally reduce the sensitivity and detection efficacy of the one-tube RPA-Cas13a technique. In the conventional two-step SHERLOCK test, transcription is separated from the RPA amplification reaction, allowing complete independence and unrestricted access to the use of their dsDNA templates (Hu et al., 2022). Additionally, the efficiency of RPA amplification and Cas13a’s cutting capability are the primary determinants influencing RPA-Cas13a’s sensitivity (Gootenberg et al., 2017).

In addition, the one-tube and two-step RPA-Cas13a detection methods reported in this study demonstrated strong specificity (Figure 7), and these two methods can reliably detect real samples (Figure 8). Using recombinase-aided amplification SHERLOCK (RAA-SHERLOCK) with a lateral-flow report, the African swine fever virus (ASFV) was successfully identified and this assay demonstrated excellent ASFV specificity, no reactivity with other swine viruses, and 100% concordance with the PCR data (Wei et al., 2022). To detect smoke mold, this two-step RPA-SHERLOCK method is also used. Compared to other molds, only the genes of tobacco mold and the plasmid DNA containing target sequences can be detected with higher specificity (Li et al., 2022). There was no discernible difference between the PCR and CRISPR methods (Li et al., 2022). In this study, we also found that the typical PCR method showed identical results with RPA-Cas13a methods in the detection of actual samples (Figure 8). Finally, since fluorescence detection is frequently more sensitive than LFD detection (Wang et al., 2020), the fluorescent technique developed in this work has a LoD of 1–10 aM (6× 10^−2^−6× 10^−1^ copies/μL; Figure 9), which is greater than LFD detection. The Ebola and Lassa virus of LASV-II was identified using the same crRNA, which had a sensitivity of 10 copies/μL with fluorescence and 100 copies/μL with LFD (Barnes et al., 2020). In fluorescence assay of another Ebola and Lassa virus of LASV-IV in the same research, the LoD was 100 copies/μL, whereas the LFD had a LoD of 1,000 copies/μL (Barnes et al., 2020). Similar phenomena were also found in the detection of dengue virus (Gootenberg et al., 2018), Porcine Circovirus Type 4 (Wang et al., 2023), SARS-CoV-2 (Su et al., 2022), epidemic diarrhea virus (PEDV; Yin et al., 2022), and hepatitis B virus (Tian et al., 2023). Even while LFD’s sensitivity is slightly lower, it can evaluate detection findings directly with the human eye and eliminates the need for fluorescence detection instruments, simplifying the equipment required. Furthermore, LFD can suit the needs of rapid pathogen detection and drug resistance gene identification because its detection period takes less than an hour.

However, in this study, the RPA-Cas13a-LFD detection method of *P. aeruginosa* DNA is only appropriate for the two-step RPA-Cas13a (Figure 9). The one-tube SHERLOCK formulation is an extremely complex and packed molecular system with numerous enzymes in its reaction components (Li H. et al., 2023). For the RPA reaction, a polymer known as polyethylene glycol (PEG) is required for the RPA reaction, which creates a crowded and extremely sticky environment for the RPA protease, interfering with the flow of other components in the experiments and being detrimental for other enzyme activities. Although the higher viscosity of the reaction solution has no effect on the fluorescent signal rising speed, PEG has impeded the flow of different components within the test strip, significantly impacting the one-step lateral flow dipstick method (Arizti-Sanz et al., 2022). To reduce viscosity, some groups employed one lyophilized pellet from the DNA constant temperature rapid amplification kit, which yielded approximately five individual reactions (Miao et al., 2023). In addition, a novel one-tube method SHERCOK was also exploited to deal with the viscosity (Xiong et al., 2022; Liu et al., 2023). Although this method reduces the risk of sample contamination and maintains detection sensitivity (Xiong et al., 2022; Liu et al., 2023), it is essentially a two-step method of SHERCOK in a strict sense. In subsequent work, we will investigate strategies to overcome the problems given by enzyme incompatibility and the crowding effect of one-tube molecular detection, thereby making the one-step RPA-Cas13a determination method a simple, dependable, and stable diagnostic tool.

## 5 Conclusion

In this study, *mexX* gene of *P. aeruginosa* was successfully detected using a one-tube and two-step RPA-Cas13a technique with reasonable specificity and sensitivity as well as lower LoD. Meanwhile, temperature, reaction time, RPA primer concentration, crRNA type and concentration all have effects on these two detection methods. In addition, a lateral flow strip measurement was also established to make this RPA-Cas13a nucleic acid detection platform rapid, affordable, and visually appealing. These platforms also demonstrate that they can detect target genes swiftly at a constant temperature of 39°C, indicating their potential for instantaneous detection. Overall, these platforms eliminate the requirement for specialized detection instruments while also making the process of detecting bacterial resistance genes easier, indicating a promising prospect for monitoring bacterial resistance on site and preventing the spread of drug-resistant microorganisms on time. However, the nucleic acid in bacterial sample must be extracted prior to detection, which necessitates the use of centrifuges and other equipment, complicating the detection procedure. Therefore, the primary focus of future research on this method should be on sample front processing technology to allow rapid environmental sample detection.

## Data availability statement

The original contributions presented in the study are included in the article/Supplementary material, further inquiries can be directed to the corresponding authors.

## Author contributions

X-XZ: Writing – original draft, Investigation, Methodology. Y-SW: Formal analysis, Methodology, Writing – review & editing. S-JL: Methodology, Resources, Writing – review & editing. R-QP: Methodology, Resources, Writing – review & editing. XW: Methodology, Resources, Writing – review & editing. HP: Data curation, Methodology, Writing – review & editing. Q-SS: Supervision, Visualization, Writing – review & editing. GZ: Formal analysis, Supervision, Validation, Writing – review & editing. X-BX: Supervision, Visualization, Writing – review & editing. JW: Supervision, Validation, Visualization, Writing – review & editing.

## Footnotes

^1^ http://www.ncbi.nlm.nih.gov/

^2^ https://cas13design.nygenoMe.org/

## Supplementary material

Supplementary Table 1 The related information of actual samples from industrial settings.
